# Minimally Invasive Aortic Valve Replacement for High-Risk Populations: Transaxillary Access Enhances Survival in Patients with Obesity

**DOI:** 10.3390/jcm13216529

**Published:** 2024-10-30

**Authors:** Ali Taghizadeh-Waghefi, Asen Petrov, Sebastian Arzt, Konstantin Alexiou, Klaus Matschke, Utz Kappert, Manuel Wilbring

**Affiliations:** 1 Faculty of Medicine Carl Gustav Carus, TU Dresden, 01307 Dresden, Germany; 2 Center for Minimally Invasive Cardiac Surgery, University Heart Center Dresden, 01307 Dresden, Germany

**Keywords:** aortic valve replacement, minimally invasive surgery, transaxillary, MICLAT-S, obesity, BMI, sternum-sparing

## Abstract

**Background/Objectives**: Minimally invasive cardiac surgery is often avoided in patients with obesity due to exposure and surgical access concerns. Nonetheless, these patients have elevated periprocedural risks. Minimally invasive transaxillary aortic valve surgery offers a sternum-sparing “nearly no visible scar” alternative to the traditional full sternotomy. This study evaluated the clinical outcomes of patients with obesity compared to a propensity score-matched full sternotomy cohort. **Methods**: This retrospective cohort study included 1086 patients with obesity (body mass index [BMI] of >30 kg/m^2^) undergoing isolated aortic valve replacement from 2014 to 2023. Two hundred consecutive patients who received transaxillary minimally invasive cardiac lateral surgery (MICLAT-S) served as a treatment group, while a control group was generated via 1:1 propensity score matching from 886 patients who underwent full sternotomy. The final sample comprised 400 patients in both groups. Outcomes included major adverse cardio-cerebral events, mortality, and postoperative complications. **Results**: After matching, the clinical baselines were comparable. The mean BMI was 34.4 ± 4.0 kg/m^2^ (median: 33.9, range: 31.0–64.0). Despite the significantly longer skin-to-skin time (135.0 ± 37.7 vs. 119.0 ± 33.8 min; *p* ≤ 0.001), cardiopulmonary bypass time (69.1 ± 19.1 vs. 56.1 ± 21.4 min; *p* ≤ 0.001), and aortic cross-clamp time (44.0 ± 13.4 vs. 41.9 ± 13.3 min; *p* = 0.044), the MICLAT-S group showed a shorter hospital stay (9.71 ± 6.19 vs. 12.4 ± 7.13 days; *p* ≤ 0.001), lower transfusion requirements (0.54 ± 1.67 vs. 5.17 ± 9.38 units; *p* ≤ 0.001), reduced postoperative wound healing issues (5.0% vs. 12.0%; *p* = 0.012), and a lower 30-day mortality rate (1.5% vs. 6.0%; *p* = 0.031). **Conclusions**: MICLAT-S is safe and effective. Compared to traditional sternotomy in patients with obesity, MICLAT-S improves survival, reduces postoperative morbidity, and shortens hospital stays.

## 1. Introduction

Obesity poses a significant challenge for cardiac surgery, particularly in the context of the current trend toward minimizing surgical trauma and physiological deterioration through minimally invasive techniques. Several publications have reported that the complexities associated with minimally invasive procedures can become a limiting factor for their application in cardiac surgery. Consequently, partly depending on the degree of obesity, patients are often excluded or deselected from these innovative approaches, as obesity is considered a relative contraindication [1,2].

The global prevalence of obesity has reached epidemic proportions. It continues its upward trajectory across nations with varying economic statuses; consequently, the World Health Organization has noted that adult obesity has more than doubled since 1990, while adolescent obesity has even quadrupled [3]. Currently, the prevalence of obesity worldwide is at its highest recorded level and continues to rise [4]. This global obesity epidemic represents a growing challenge for healthcare systems worldwide, which is also reflected in the increasing number of patients with obesity among the cardiac surgical patient population [5]. Furthermore, the demographic shift toward an older population, coupled with an increase in age-related degenerative diseases, has led to a higher prevalence of obesity among patients diagnosed with severe aortic stenosis (AS) [6,7]. Consequently, these patients are increasingly undergoing either surgical aortic valve replacement (SAVR) or transcatheter aortic valve implantation (TAVI) [7]. However, patients with obesity may face additional risks due to their heightened risk profiles and anatomical peculiarities during conventional surgical procedures performed via sternotomy. These patients are at a higher risk of developing deep sternal wound infections following cardiac surgery, along with other complications, such as wound dehiscence, the requirement for prolonged ventilation, and longer hospital stays [8,9,10]. Nonetheless, mortality following SAVR in patients with obesity remains a contentious topic in the literature, as some studies have reported reduced mortality after cardiac surgery [11]. This unanticipated and seemingly contradictory phenomenon is referred to as the “obesity paradox” [11,12,13]. This paradox may be explained primarily by selection bias. In risk stratification for elective cardiac surgery, individuals with a high body mass index (BMI) but without metabolic syndrome are more likely to be considered for surgery than those with a higher risk of obesity-related complications [14,15]. Notably, the obesity paradox appears to disappear when evaluating long-term postoperative results, such as the late development of cardiometabolic diseases and the early onset of sternal dehiscence and mediastinitis after sternotomy [16,17,18,19]. However, the paradox stands in contrast to findings that identify obesity as an independent predictor of higher hospital mortality [20].

Amid the abovementioned controversies and uncertainties, minimally invasive cardiac surgery (MICS) has gained recognition as a viable alternative aimed at reducing complications and risks, even in high-risk groups. However, it remains unclear whether this promise is substantiated, despite recent advances in MICS. Previous studies have highlighted ongoing uncertainties about the benefits for obese patients, as a smaller incision may result in inadequate surgical field exposure and extended procedure times [21,22]. Furthermore, a large proportion of previous studies on MICS have focused primarily on patients with a lower BMI or an average risk profile. This study sought to fill this gap by investigating the outcomes of MICS–aortic valve replacement (AVR) in obese patients, a group that may be at an elevated risk during surgical procedures. Given the rising rates of obesity and the increasing population of older adults with aortic stenosis, it is essential to establish safe and effective treatment options for these vulnerable groups of patients. In this regard, evaluating MICS as a promising option for patients with obesity and AS can provide valuable insights and potentially influence clinical practice. To date, MICS has not shown any clear survival advantages in the broader population of cardiac surgery patients, likely due to the influence of selection bias. This study evaluated the outcomes of MICS in obese patients, compared to a propensity score-matched cohort who underwent sternotomy.

## 2. Patients and Methods

### 2.1. Inclusion and Exclusion Criteria

The main aim of this study was to assess adult patients with obesity undergoing SAVR, specifically comparing those treated with the transaxillary minimally invasive cardiac lateral surgery (MICLAT-S) approach to those treated with a traditional full sternotomy. Obesity was classified as having a BMI greater than 30 kg/m^2^. Patients were excluded if they had undergone additional concomitant procedures, had a history of active or recent endocarditis, had previous cardiac surgeries, or did not meet the defined BMI criterion of over 30 kg/m^2^.

### 2.2. Study Design and Ethical Statement

The data for this analysis were gathered retrospectively from electronic health records. The primary outcomes of interest were major adverse cardio-cerebral events (MACCEs), including perioperative myocardial infarction, ischemic stroke, and 30-day mortality. Secondary outcomes included postoperative complications and morbidities. The study protocol received approval from the local ethics committee (EK—Nr. 28092012).

### 2.3. Patient Population

**Figure 1:**
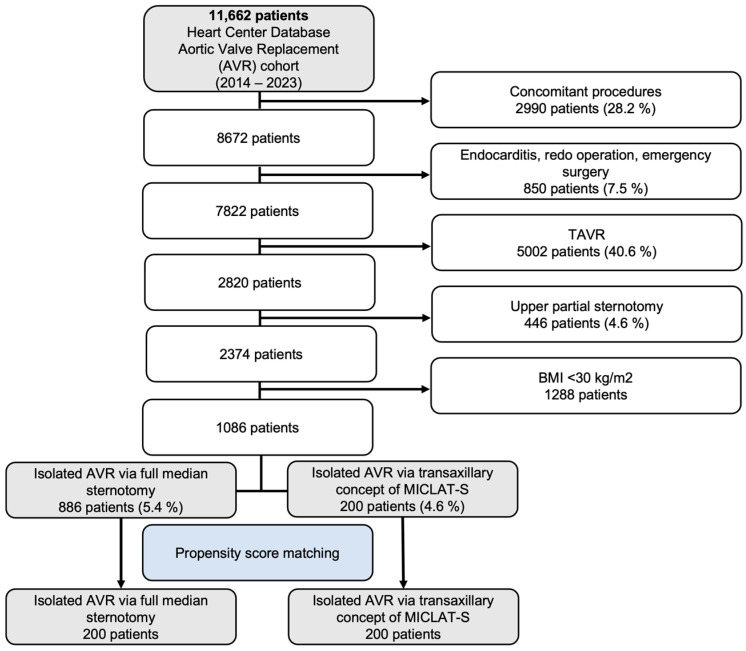
Flow diagram of the study population. TAVR, transcatheter aortic valve replacement; AVR, aortic valve replacement; MICLAT-S, minimally invasive cardiac lateral surgery.

Between 2014 and 2023, a total of 11,662 patients were included in the Heart Center Database for AVR. Out of these, 2990 patients (28.2%) who underwent concomitant procedures were excluded, along with 850 patients (7.5%) who had endocarditis, redo operations, or emergency surgeries. An additional 5002 patients (40.6%) who underwent transcatheter aortic valve implantation (TAVI) were also excluded, followed by 446 patients (4.6%) who received an upper partial sternotomy and 1288 patients with a body mass index (BMI) below 30 kg/m^2^. This left a final cohort of 1086 patients meeting the inclusion criteria. These patients were then divided into two groups: 886 patients (5.4%) who underwent isolated AVR via full median sternotomy and 200 patients (4.6%) who had isolated AVR via the transaxillary MICLAT-S approach. Propensity score matching was applied, resulting in 200 patients in each group for further analysis. The patient selection process is illustrated in the flowchart in Figure 1.

### 2.4. Involved Surgeons

All MICS-AVR procedures were mainly carried out by four highly experienced surgeons, each with extensive expertise in adult cardiac surgery. Their individual experience with procedures involving extracorporeal circulation ranged from over 500 to 6500 cases. Additionally, based on the structured modular training program and the developments within our surgical team, 64% of the surgical staff were actively involved in performing either the entire procedure or specific parts of it. This involvement stemmed from a well-organized educational approach, where surgical training for residents is divided into linear modules, covering all aspects from femoral vessel preparation to aortic valve surgery. Despite potential confounding factors, such as the transition from selected to all-comer patients and the increased focus on education, these changes have been integrated into the program, resulting in a high level of participation across the surgical team.

### 2.5. Sternum-Sparing Transaxillary Concept of MICLAT-S

Since the surgical technique for AVR via median sternotomy is well known, it will not be elaborated upon further herein. With the establishment of the MICLAT-S concept at the end of 2019, the year in which the first MICLAT-S approach was implemented, all other MICS approaches—such as upper partial hemisternotomy and right anterolateral thoracotomy—were displaced and outperformed alongside sternotomy. Consequently, by the final year of the study, 97.8% of the AVR procedures were performed using the MICLAT-S approach [23].

**Figure 2:**
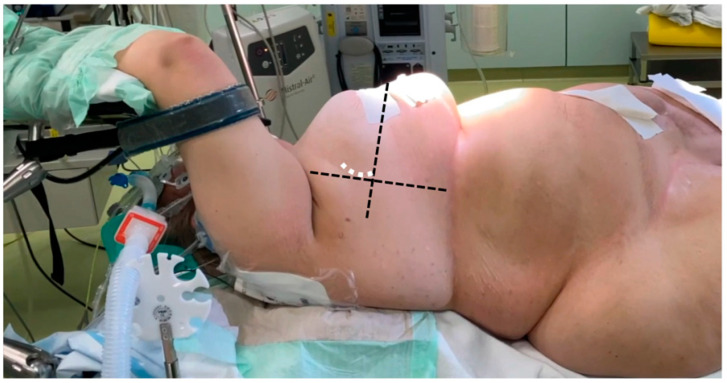
Javelin thrower’s position in a female patient with obesity (body mass index = 53.9 kg/m^2^). The horizontal dashed line marks the anterior axillary line, while the orthogonal line originates from the mid-sternum (excluding the xiphoid). The white dashed line indicates the incision line.

For preoperative planning, the anatomical details of the patients were assessed through electrocardiography-gated CT angiography, covering the thoracic, abdominal, and pelvic regions. All patients underwent general anesthesia, and intraoperative transesophageal echocardiography (TEE) was routinely used as the imaging standard, regardless of the surgical technique. In all sternum-sparing MICLAT-S procedures, a double-lumen tube was employed for intubation to allow for single-lung ventilation. Additionally, a temporary transvenous pacing wire was placed via a percutaneous sheath. The femoral vessels were accessed using a conventional surgical cut-down, and extracorporeal circulation was initiated following TEE-guided cannulation. A percutaneous approach for femoral cannulation was only performed if clinical signs of inflammation in the groin area were apparent. Common to all MICS-AVR procedures was the insertion of an antegrade cardioplegia cannula into the ascending aorta, along with the placement of a left ventricular vent line through the right superior pulmonary vein. The method for AVR access via the transaxillary MICLAT-S approach has been described in previous publications. In brief, the patient was positioned supine, with the right side of the chest elevated on two pillows and the right arm resting on the arm support, a position known as the javelin thrower’s position (Figure 2). A 5 cm incision was created along the right anterior axillary line, followed by the dissection of the serratus anterior and intercostal muscles to enter the third or fourth intercostal space [24,25]. A video of our AVR procedure using the MICLAT-S technique is available as Supplementary Materials.

### 2.6. Prosthesis Choice

The choice of prosthesis was largely influenced by the surgeon’s preference, especially for MICS-AVR procedures. In these cases, comprehensive anatomical information was obtained through a high-resolution, full cardiac cycle CT scan, following the TAVI-CT protocol, covering the thorax, abdomen, and pelvis. This scan provided important preoperative data, including measurements such as the distance from the annular plane to the chest wall, the size of the aortic annulus, and the anticipated valve size. These measurements played a key role in the surgeon’s decision-making process. With growing evidence supporting the durability of rapid deployment valves (RDVs), their use was not restricted [26,27,28].

### 2.7. Statistical Analysis

The Shapiro–Wilk test was used to check if continuous variables followed a normal distribution. For those that did, a *t*-test was applied, while the Mann–Whitney U test was used for non-normally distributed variables and those on ordinal scales. Fisher’s exact test and the chi-square test were employed to analyze dichotomous and other categorical variables, respectively.

Propensity score matching was conducted to address baseline imbalances between the groups, considering factors such as age, sex, EuroSCORE II, BMI, left ventricular ejection fraction (LVEF), atrial fibrillation, peripheral vascular disease, chronic kidney disease, and chronic lung disease. Since corrections for type I errors were not implemented for multiple comparisons, the inferential statistical results serve a descriptive purpose, with significance indicated by a local *p*-value below 0.05, without implying an error rate under 5%. All statistical analyses were performed using the open-source R software (version 4.4.1).

## 3. Results

### 3.1. Baseline Patient Characteristics

From January 2014 to February 2023, a total of 1086 patients underwent surgery, with 886 receiving a sternotomy and 200 undergoing MICLAT-S. Several significant differences in the baseline demographic and clinical characteristics were observed between the two groups. The sternotomy group tended to be older (*p* = 0.034), while the MICLAT-S group more frequently had atrial fibrillation (*p* = 0.021). Additionally, the sternotomy group showed a higher prevalence of diabetes mellitus (*p* = 0.038) and peripheral arterial occlusive disease (*p* = 0.004). The MICLAT-S group was significantly more likely to have had a transient ischemic attack (*p* < 0.001). However, there were no significant differences concerning the BMI, LVEF, or EuroSCORE II. A significant difference was noted in the prevalence of pulmonary arterial hypertension, which was considerably more common in the sternotomy group (*p* < 0.001).

Propensity scores, based on the defined variables, were applied to match 200 patients from each group within the full cohort, ensuring comparable baseline characteristics between them. After matching, there was clear alignment in the baseline characteristics across both groups. Table 1 provides an overview of the baseline characteristics for both the unmatched and propensity score-matched groups.

### 3.2. Unadjusted Outcomes

#### 3.2.1. Procedural and Intraoperative Data

**Figure 3:**
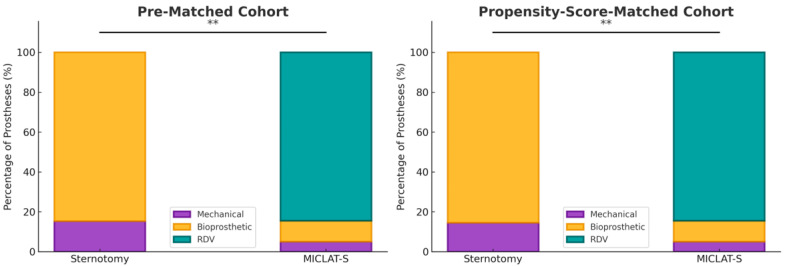
Distribution of the type of aortic valve prostheses implanted within each group. MICLAT-S, minimally invasive cardiac lateral surgery; RDV, rapid deployment valve; ** *p* ≤ 0.01.

**Figure 4:**
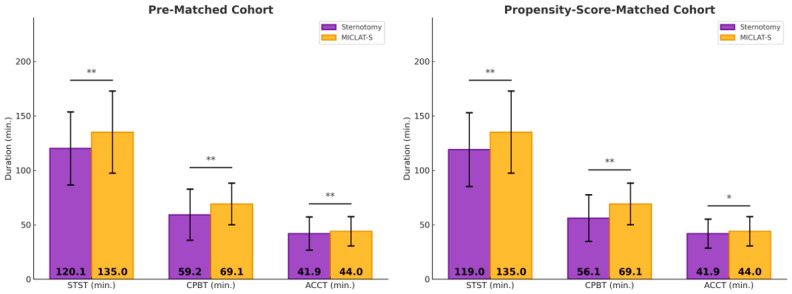
Surgical times. * *p* < 0.05 between groups; MICLAT-S, minimally invasive cardiac lateral surgery; min, minute; STST, skin-to-skin time; CPBT, cardiopulmonary bypass time; ACCT, aortic cross-clamp time; * *p* ≤ 0.05; ** *p* ≤ 0.01.

Among the pre-matched cohort, the prosthesis size was significantly larger in the MICLAT-S group (24.2 ± 2.1 mm) than in the sternotomy group (23.2 ± 1.8 mm; *p* ≤ 0.001; Table 2). Regarding the prosthesis type, RDVs were used in 81.9% of the patients in the MICLAT-S group, while none were used in the sternotomy group. Bioprosthetic and mechanical valves were significantly more commonly used in the sternotomy group (84.7% vs. 13.4%; *p* ≤ 0.001; 15.3% vs. 4.7%, respectively; Figure 3; Table 2). The MICLAT-S group also exhibited a significantly longer skin-to-skin time (STST) (135.0 ± 37.7 vs. 120.1 ± 33.6 min; *p* ≤ 0.001), cardiopulmonary bypass time (CPBT) (69.1 ± 19.1 vs. 59.2 ± 23.5 min; *p* ≤ 0.001), and aortic cross-clamp time (ACCT) (44.0 ± 13.4 vs. 41.9 ± 15.2 min; *p* = 0.044; Figure 4; Table 2).

#### 3.2.2. Postoperative Outcomes, Morbidity, and Mortality

Among the pre-matched cohort, significant differences were observed in several key postoperative outcomes between the MICLAT-S and sternotomy groups. A ventilation time of ≤12 h was significantly more common in the MICLAT-S group than in the sternotomy group (90.5% vs. 26.2%; *p* ≤ 0.001). Regarding the intensive care unit (ICU) stay, the MICLAT-S group demonstrated a shorter duration, with 70.5% of the patients staying ≤24 h, compared to 57.7% in the sternotomy group (*p* = 0.006). Likewise, the overall hospital stay was notably shorter in the MICLAT-S group (9.71 ± 6.19 vs. 12.6 ± 8.60 days; *p* ≤ 0.001). The MICLAT-S group required significantly fewer packed red blood cell transfusions, with an average of 0.54 ± 1.67 units, compared to 4.02 ± 7.37 units in the sternotomy group (*p* ≤ 0.001). Although the incidence of acute kidney injury (AKI) stage III or continuous veno-venous hemofiltration (CVVH) was lower in the MICLAT-S group (2.0% vs. 5.0%), this difference did not reach statistical significance (*p* = 0.097). However, the re-exploration rates were significantly higher in the MICLAT-S group (6.5% vs. 3.2%; *p* = 0.043). There was no significant difference between the groups in the incidence of wound healing complications and postoperative delirium. The incidence of both ischemic and disabling strokes was comparable between the groups. Additionally, the 30-day mortality rate was lower in the MICLAT-S group, although the difference was not statistically significant (1.5% vs. 3.4%; *p* = 0.240). Lastly, MACCEs were significantly less common in the MICLAT-S group than in the sternotomy group (2.0% vs. 5.1%; *p* = 0.028). The results for the postoperative parameters for the pre-matched cohort can be found in Table 3.

### 3.3. Propensity Score-Matched Cohort

To mitigate the influence of potential confounding variables, we applied propensity score matching between the two groups, resulting in 400 patients (200 matched pairs) for further analysis.

#### 3.3.1. Adjusted Procedural and Intraoperative Data

Among the propensity score-matched cohort, the prosthesis size remained significantly larger in the MICLAT-S group (24.1 ± 2.0 vs. 23.7 ± 1.9 mm; *p* = 0.041; Table 2). The prosthesis type distribution remained consistent with the pre-matched cohort. RDVs were used in 84.5% of the patients in the MICLAT-S group, while none were used in the sternotomy group. Bioprosthetic and mechanical valves were significantly more commonly used in the sternotomy group (85.5% vs. 10.5%; *p* ≤ 0.001; 14.5% vs. 5.0%, respectively; Figure 3; Table 2).The MICLAT-S group continued to show a significantly longer STST (135.0 ± 37.7 vs. 119.0 ± 33.8 min; *p* ≤ 0.001), CPBT (69.1 ± 19.1 vs. 56.1 ± 21.4 min; *p* ≤ 0.001), and ACCT (44.0 ± 13.4 vs. 41.9 ± 13.3 min; *p* = 0.044; Figure 4; Table 2).

#### 3.3.2. Adjusted Postoperative Outcomes, Morbidity, and Mortality

**Figure 5:**
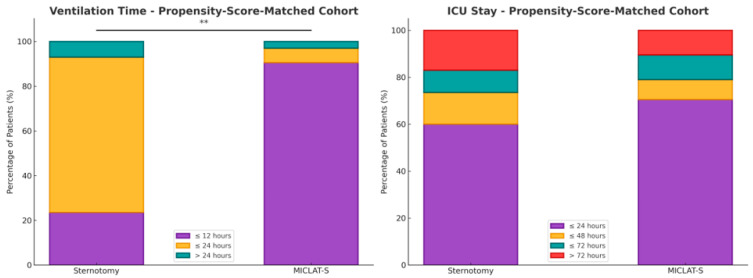
Two stacked bar charts present the percentage distribution of patients in the propensity score-matched cohort for both the ventilation time (on the left) and ICU stay (on the right). Each bar represents the percentage of patients in each group who experienced different durations of ventilation (min) or ICU stays (day). The stacked bars for both the sternotomy and MICLAT-S groups total 100%, representing the total distribution of patients across the different time intervals; ** *p* ≤ 0.01.

**Figure 6:**
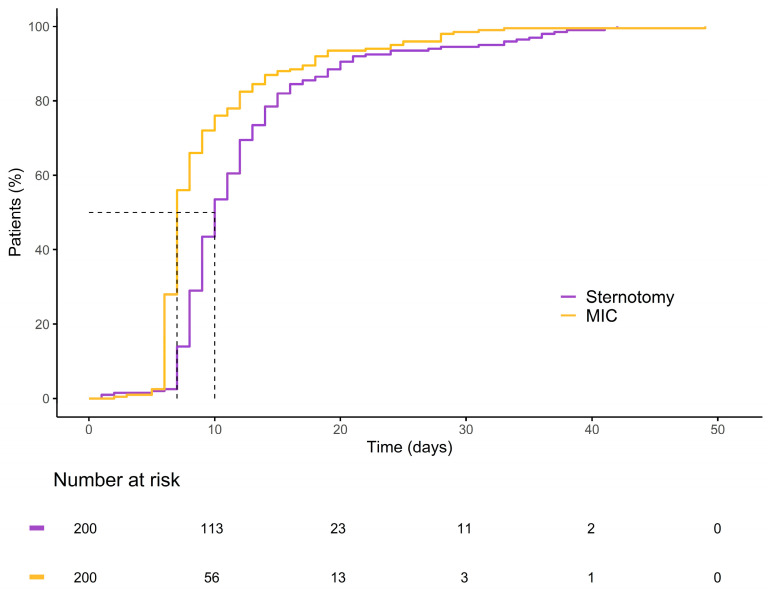
Kaplan–Meier curve illustrating the cumulative probability of patients remaining hospitalized over time for each group.

Within the matched cohort, the MICLAT-S group continued to demonstrate significant advantages. The proportion of patients requiring ventilation for ≤12 h remained significantly larger in the MICLAT-S group (90.5%) than in the sternotomy group (23.5%) (*p* ≤ 0.001; Figure 5). The trend of shorter ICU stays seen in the unmatched cohort did not achieve statistical significance in the matched cohort, although it did show a statistical tendency (70.5% of the patients in the MICLAT-S group vs. 60.0% of those in the sternotomy group stayed ≤24 h; *p* = 0.068; Figure 5). However, the overall hospital stay, which was significantly shorter in the MICLAT-S group in the unmatched cohort, remained significantly reduced among the matched cohort (9.71 ± 6.19 vs. 12.4 ± 7.13 days; *p* ≤ 0.001; Figure 6). In alignment with the unmatched cohort findings, the MICLAT-S group among the matched cohort required significantly fewer transfusions of packed red blood cells (0.54 ± 1.67 vs. 5.17 ± 9.38 units; *p* ≤ 0.001). The lower incidence of AKI stage III or postoperative-onset CVVH was confirmed in the matched cohort (2.0% vs. 9.0%; *p* = 0.022). The re-exploration rates, which were significantly higher in the MICLAT-S group among the unmatched cohort, remained elevated among the matched cohort (6.5% vs. 2.5%) but did not reach statistical significance (*p* = 0.089). Conversely, the trend of reduced wound healing complications in the MICLAT-S group continued and became statistically significant in the matched cohort (5.0% vs. 12.0%; *p* = 0.012). Although postoperative delirium syndrome occurred more frequently in the MICLAT-S group in both the unmatched and matched cohorts, the difference was not statistically significant in the matched cohort (20.0% vs. 13.0%; *p* = 0.0794). Notably, the difference in the 30-day mortality rate, which was not statistically significant among the unmatched cohort, became significant after propensity score matching, with the MICLAT-S group showing a lower rate (1.5% vs. 6.0%; *p* = 0.031). Moreover, the propensity score matching reinforced the significantly lower incidence of MACCEs in the MICLAT-S group (2.0% vs. 7.5%; *p* = 0.003). Postoperative outcomes for the propensity score-matched cohort are detailed in Table 3.

## 4. Discussion

Minimally invasive techniques for AVR have evolved significantly over the past three decades, with sternum-sparing approaches emerging even before the introduction of the upper partial hemisternotomy. In the 1990s, pioneering efforts by Rao and Kumar (1993) and Cosgrove and Sabik (1996) laid the groundwork for sternum-sparing approaches in MICS-AVR [29,30]. Notably, Cosgrove advanced the field by reducing thoracic trauma through the incorporation of femoral cannulation in MICS-AVR [30]. The introduction of MICS-AVR via upper partial hemisternotomy by Svensson in 1997 marked another milestone [31]. However, before the widespread clinical adoption of TAVI, MICS-AVR accounted for less than 5% of all cases in Germany [28]. The growing awareness of and emphasis on reducing surgical invasiveness, spurred by the advent of TAVI, ultimately led to a renaissance for sternum-sparing techniques, exemplified by the development of RAT by Lamelas in 2015 [32]. This approach was further refined with the introduction of the endoscopically guided right anterolateral thoracotomy in 2020 [33].

After nearly 30 years of development, the current state of cardiac surgery in the treatment of aortic valve pathologies presents a rather sobering conclusion. Despite innovations, the adoption of sternum-sparing techniques remains notably low, and they are not even mentioned in the annual German Heart Surgery Reports [29]. Additionally, while the upper partial hemisternotomy for AVR remains the most commonly used minimally invasive technique, its adoption has increased but still remains below 50%; it is currently at 43.2% [34]. This suggests that most patients undergoing treatment for aortic valve pathologies still undergo a conventional full sternotomy. Notably, the adoption rate of minimally invasive procedures for isolated AVR is low, and there is ongoing hesitancy regarding their use. A plausible explanation is that the minimally invasive techniques developed thus far carry an inherent limitation, restricting their applicability to a highly selective patient cohort [35]. This high degree of selectivity hinders the establishment of an evidence-based foundation for the application of minimally invasive techniques across the broader all-comer patient population. It also creates clinical bias by selecting lower-risk patients for minimally invasive procedures, which in turn leads to insufficient statistical power to show a significant reduction in mortality compared to the median sternotomy approach; this is particularly relevant as no studies to date have demonstrated a clear mortality benefit for MICS in the broader cardiac surgery population [21,36,37,38,39].

To the best of our knowledge, our study is the first to present evidence from a substantial cohort of 1086 patients, showing that individuals with obesity and undergoing MICS-AVR experience significantly better survival and reduced postoperative morbidity than a propensity score-matched sternotomy cohort. The main results of this study are as follows:

- Combined MACCEs were less frequently observed in the MICLAT-S group;
- The postoperative 30-day mortality rate was significantly lower in the MICLAT-S group;
- The MICLAT-S group had a shorter median hospital stay;
- The incidence of postoperative impaired wound healing was significantly lower in the MICLAT-S group;
- The MICLAT-S group needed fewer transfusions of blood products.

Patients with obesity have been excluded from major reports on minimally invasive valve surgery, which is why comparative studies are scarce. The right anterolateral thoracotomy, as a sternum-sparing approach for AVR, has so far only been examined in small case series comparing valve surgeries to sternotomy or in comparative studies with limited patient numbers [40,41]. Further studies have investigated the upper partial hemisternotomy as a minimally invasive option, comparing it to a median sternotomy in obese patients [42,43,44,45,46,47,48]. However, these studies have not provided evidence of the superiority of MICS techniques over conventional methods in terms of mortality for isolated AVR. Among the defined primary endpoints in the present study, while the incidence of perioperative stroke and myocardial infarction was comparable between the two groups, that of both 30-day mortality and MACCEs was significantly lower in the MICLAT-S group than in the sternotomy group. These findings confirm that the transaxillary concept of MICLAT-S, as a sternum-sparing MICS-AVR technique, outperforms the conventional full median sternotomy for AVR in patients with obesity. Notably, throughout the study period, the selection of patients for the MICLAT-S approach was entirely independent of anthropometric factors.

In terms of hospital resource utilization, the MICLAT-S group demonstrated clear superiority. The MICLAT-S group experienced notably shorter ventilation times and overall hospital stays compared to the control group. After matching, only a statistical trend was observed regarding shorter ICU stays in the MICLAT-S group. This observation is consistent with the findings of other studies that compared MICS procedures to sternotomy in patients with obesity for isolated AVR [40,43,44,45,46,49,50]. A possible explanation for the shorter hospital stays could be that the MICLAT-S technique, which is bone-sparing and avoids affecting the shoulder girdle, may enable earlier patient mobilization and accelerated recovery, leading to quicker patient independence and earlier discharge. However, this hypothesis requires further validation in future studies. Moreover, consistent with previous reports, the MICLAT-S group required significantly fewer perioperative transfusions of packed red blood cells [40,42,45,46,51].

One drawback of the minimally invasive procedure was the extended procedural duration, which included a longer surgical time, CPBT, and cross-clamp time. However, this did not have any clinically apparent impact on the primary endpoints or other postoperative morbidities. On the contrary, the need for perioperative transfusions and the incidence of acute renal failure and postoperative new-onset dialysis were lower in the MICS-AVR group. While the lower incidence of acute renal failure in the MICLAT-S group, despite longer procedural times, might seem surprising, the most plausible explanation lies in the significantly fewer complications and reduced transfusion requirements in the minimally invasive group—both of which are key contributors to the development of renal failure [40,52].

RDVs were exclusively implanted in the MICLAT-S group, consistent with previous reports and likely explaining the implantation of larger valve prostheses in this cohort [53].

In the MICLAT-S group, seven patients (1.6%) underwent conversion to sternotomy. However, none of these conversions were attributed to inadequate exposure of the aortic valve.

As anticipated, the occurrence of thoracic wound healing complications was significantly higher in the sternotomy group than in the MICLAT-S group. The groups differed not only in the incidence but also in the location of such disorders. In the MICLAT-S group, wound healing complications predominantly arose at the groin (60%, *n*/*N* = 6/10) and were less common in the thoracic area (40%, *n*/*N* = 4/10), whereas, in the control group, they were exclusively localized to the sternum. Notably, 45.8% (*n*/*N* = 11/24) of the patients with sternal wound healing disorders in the control group developed consequent mediastinitis. In contrast, no cases of mediastinitis were observed in the MICLAT-S group. Among the cases of local wound healing disorders in the groin, 66.7% (*n*/*N* = 4/6) were primarily attributed to the formation of a lymph fistula, while the remaining cases had a primary infection of the groin wound.

**Figure 7:**
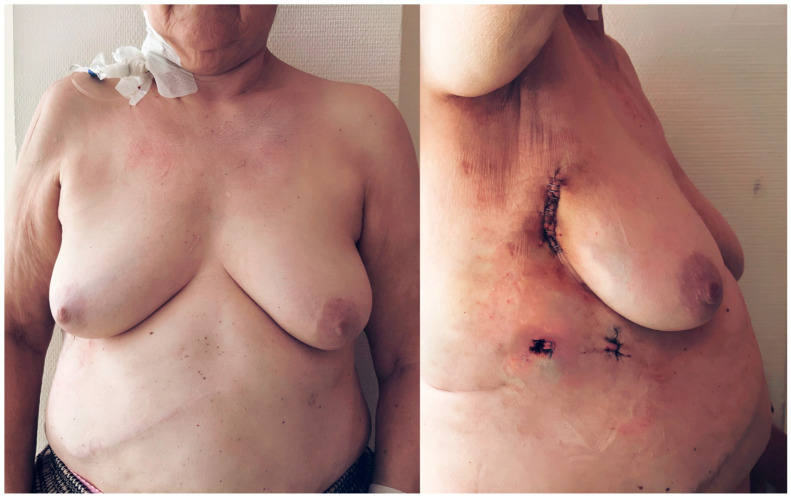
Postoperative cosmetic outcomes of minimally invasive cardiac lateral surgery in a female patient with obesity (body mass index of 31.1 kg/m^2^). The images illustrate the minimally invasive nature of the procedure. The sternum remains intact, and the incisions are placed in the right anterior axillary line, minimizing visible scarring, particularly in female patients with a higher body mass index. The right image demonstrates the primary surgical incision, contributing to enhanced cosmetic outcomes and more comfortable postoperative recovery.

Finally, the cosmetic outcomes of the transaxillary concept of MICLAT-S were particularly satisfactory, especially in the female patients with obesity, due to the minimized visibility of scars and the sternum-sparing nature of the procedure (Figure 7).

## 5. Conclusions

The results underscore the potential of the holistic transaxillary concept of MICLAT-S as a viable, more broadly applicable alternative to traditional sternotomy, particularly in patients with obesity. Given the limited research on minimally invasive techniques, especially sternum-sparing procedures, this study provides crucial evidence that could pave the way for the broader adoption and further exploration of these approaches in this challenging patient population. Furthermore, the results of this study offer a strong basis for the hypothesis that minimally invasive procedures, when extended beyond the confines of highly selective patient cohorts and applied as a therapeutic strategy for the broader all-comer population, have the potential to confer significant advantages, even in high-risk and high-morbidity patient groups. Consequently, the overarching goal of minimally invasive techniques should not be limited by the specific method of implementation but should instead focus on making them accessible to the entire spectrum of cardiac surgical patients. Therefore, it is not the patient who must be deemed suitable for the MICS approach, but rather the MICS approach that must be adapted to meet the needs of all patients. While TAVI is seeking to further expand its evidence base from high- to intermediate- and low-risk patients, MICS-AVR remains predominantly in the domain of low-risk patients. One reason that an all-comer approach has so far failed to gain widespread acceptance for MICS-AVR in cardiac surgery centers is the lack of a holistic concept. Therefore, the emerging generation of cardiac surgeons must address the areas of greatest need, breaking the paradigm of offering minimally invasive procedures only to low-risk patients and extending MICS to all risk groups, regardless of their morbidity profile. Immediate efforts should be directed toward integrating these findings into clinical practice, ensuring that the benefits of MICLAT-S are made available to a broader range of patients, thereby advancing the field of cardiac surgery as a whole.

## 6. Limitations

This study has several limitations. First, although the cohort was large, the research was conducted at a single center and was a retrospective analysis with a relatively short follow-up period. Second, the propensity score matching model may have missed unknown but potentially important risk factors and confounding variables. Third, the matching criteria were mainly chosen based on the surgical feasibility of minimally invasive AVR. Finally, the outcomes were obtained in a high-volume, specialized center, which may reduce the generalizability of the findings to wider patient populations.

## Figures and Tables

**Table 1:** Baseline characteristics.

	Pre-Matched Cohort			Propensity Score-Matched Cohort		
	Sternotomy (*n* = 886)	MICLAT-S (*n* = 200)	*p*	Sternotomy (*n* = 200)	MICLAT-S (*n* = 200)	*p*
Age (year), mean ± SD	69.0 ± 8.65	67.6 ± 8.27	*0.034* *	68.4 ± 8.25	67.6 ± 8.27	0.327
BMI (kg/m^2^), mean ± SD	33.8 ± 3.43	34.1 ± 3.76	0.205	34.6 ± 4.21	34.1 ± 3.76	0.227
Diabetes mellitus, *n* (%)	379 (42.8)	69 (35.5)	*0.038* *	75 (37.5)	69 (34.5)	0.603
Previous MI, *n* (%)	24 (2.7)	6 (3.0)	1	8 (4.0)	6 (3.0)	0.787
LVEF > 50%, *n* (%)	671 (75.7)	155 (77.5)	0.156	159 (79.5)	155 (77.5)	0.948
Atrial fibrillation, *n* (%)	107 (12.1)	37 (18.5)	*0.021* *	48 (24.0)	37 (18.5)	0.221
COPD, *n* (%)	75 (8.5)	24 (12.0)	0.152	31 (15.5)	24 (12.0)	0.384
Renal insufficiency, *n* (%)	243 (27.4)	48 (24.0)	0.368	53 (26.5)	48 (24.0)	0.645
Hemodialysis, *n* (%)	7 (0.8)	4 (2.0)	0.249	4 (2.0)	4 (2.0)	1
PAOD, *n* (%)	126 (14.2)	13 (6.5)	*0.004* **	17 (8.5)	13 (6.5)	0.570
TIA, *n* (%)	0 (0)	4 (2.0)	*<0.001* **	0 (0)	4 (2.0)	0.123
EuroSCORE II (%), mean ± SD	1.68 ± 1.24	1.83 ± 1.21	0.286	1.83 ± 1.81	1.83 ± 1.21	0.989

Bold and italic values indicate statistical significance: * *p* ≤ 0.05; ** *p* ≤ 0.01; MICLAT-S, minimally invasive cardiac lateral surgery; SD, standard deviation; BMI, body mass index; LVEF, left ventricular ejection fraction; COPD, chronic obstructive pulmonary disease; PAOD, peripheral arterial occlusive disease; TIA, transient ischemic attack.

**Table 2:** Procedural and intraoperative data.

	Pre-Matched Cohort			Propensity Score-Matched Cohort		
	Sternotomy (*n* = 886)	MICLAT-S (*n* = 200)	*p*	Sternotomy (*n* = 200)	MICLAT-S (*n* = 200)	*p*
Prosthesis size (mm), mean ± SD	23.2 ± 1.8	24.2 ± 2.1	*≤0.001* **	23.7 ± 1.9	24.1 ± 2.0	*0.041* *
STST (min), mean ± SD	120.1 ± 33.6	135.0 ± 37.7	*≤0.001* **	119.0 ± 33.8	135.0 ± 37.7	*≤0.001* **
CPBT (min), mean ± SD	59.2 ± 23.5	69.1 ± 19.1	*≤0.001* **	56.1 ± 21.4	69.1 ± 19.1	*≤0.001* **
ACCT (min), mean ± SD	41.9 ± 15.2	44.0 ± 13.4	*0.044* *	41.9 ± 13.3	44.0 ± 13.4	*0.044* *
Prosthesis type						
- Mechanical, *n* (%)	136 (15.3)	10 (5.0)		29 (14.5)	10 (5.0)	
- Bioprosthetic, *n* (%)	750 (84.7)	21 (10.5)	*≤0.001* **	171 (85.5)	21 (10.5)	*≤0.001* **
- RDV, *n* (%)	0 (0.0)	169 (84.5)		0 (0.0)	169 (84.5)	

Bold and italic values indicate statistical significance: * *p* ≤ 0.05; ** *p* ≤ 0.01; MICLAT-S, minimally invasive cardiac lateral surgery; SD, standard deviation; min, minute; STST, skin-to-skin time; CPBT, cardiopulmonary bypass time; ACCT, aortic cross-clamp time; RDV, rapid deployment valve.

**Table 3:** Postoperative morbidity and mortality.

	Pre-Matched Cohort			Propensity Score-Matched Cohort		
	Sternotomy (*n* = 886)	MICLAT-S (*n* = 200)	*p*	Sternotomy (*n* = 200)	MICLAT-S (*n* = 200)	*p*
Ventilation time (h) - ≤12, *n* (%) - ≤24, *n* (%) - >24, *n* (%)	232 (26.2) 525 (59.3) 57 (6.4)	181 (90.5) 13 (6.5) 6 (3.0)	*≤0.001* **	47 (23.5) 139 (69.5) 14 (7.0)	181 (90.5) 13 (6.5) 6 (3.0)	*≤0.001* **
Respiratory failure ^†^, *n* (%)	40 (4.5)	6 (3.0)	0.442	11 (5.5)	6 (3.0)	0.322
ICU stay (days), mean ± SD - ≤24, *n* (%) - ≤48, *n* (%) - <72, *n* (%) - >72, *n* (%)	511 (57.7) 133 (15.0) 89 (10.0) 152 (17.2)	141 (70.5) 17 (8.5) 21 (10.5) 21 (10.5)	*0.006* **	120 (60.0) 27 (13.5) 19 (9.5) 34 (17.0)	141 (70.5) 17 (8.5) 21 (10.5) 21 (10.5)	0.068
Hospital stay (days), mean ± SD	12.6 ± 8.60	9.71 ± 6.19	*≤0.001* **	12.4 ± 7.13	9.71 ± 6.19	*≤0.001* **
Transfusion of PRBCs, mean ± SD	4.02 ± 7.37	0.540 ± 1.67	*≤0.001* **	5.17 ± 9.38	0.540 ± 1.67	*≤0.001* **
AKI stage III or CVVH, *n* (%)	44 (5.0)	4 (2.0)	0.097	18 (9.0)	4 (2.0)	*0.022* *
Conversion to sternotomy, *n* (%)	N/A	7 (1.6)	N/A	N/A	7 (1.6)	N/A
Re-exploration, *n* (%)	28 (3.2)	13 (6.5)	*0.043* *	5 (2.5)	13 (6.5)	0.0888
Impaired wound healing, *n* (%)	53 (6.0)	10 (5.0)	0.707	24 (12.0)	10 (5.0)	*0.012* **
Postoperative delirium, *n* (%)	157 (17.7)	40 (20.0)	0.517	26 (13.0)	40 (20.0)	0.0794
Ischemic stroke (Rankin ≥ 2), *n* (%)	12 (1.4)	1 (0.5)	0.490	2 (1.0)	1 (0.5)	0.745
TIA, *n* (%)	9 (1.0)	1 (0.5)	0.745	2 (1.0)	1 (0.5)	0.618
PPM implantation, *n* (%)	4 (0.5)	11 (5.5)	0.446	1 (0.5)	11 (5.5)	1
Myocardial infarction, *n* (%)	4 (0.5)	0 (0.0)	0.758	1 (0.5)	0 (0)	1
30-day mortality, *n* (%)	30 (3.4)	3 (1.5)	0.240	12 (6.0)	3 (1.5)	*0.031* *
MACCE, *n* (%)	45 (5.1)	4 (2.0)	*0.028* *	15 (7.5)	4 (2.0)	*0.003* **

Bold and italic values indicate statistical significance: * *p* ≤ 0.05; ** *p* ≤ 0.01; ^†^ Primary postoperative ventilation time of ≥72 h, reintubation, and tracheotomy; MICLAT-S, minimally invasive cardiac lateral surgery; SD, standard deviation; ICU, intensive care unit; PRBC, packed red blood cell; AKI, acute kidney injury; CVVH, continuous veno-venous hemofiltration; TIA, transient ischemic attack; PPM, permanent pacemaker; N/A, not applicable.

## Data Availability

The data presented in this study are available on request from the corresponding author. The data are not publicly available due to ethical regulations.

## Supplementary Materials

The following supporting information can be downloaded at: https://zenodo.org/records/13760664; Minimally Invasive Transaxillary Approach: Aortic Valve Replacement in a Patient with BMI > 50 kg/m^2^.
